# Anti-Tumoral Effect of the Mitochondrial Target Domain of Noxa Delivered by an Engineered *Salmonella typhimurium*

**DOI:** 10.1371/journal.pone.0080050

**Published:** 2014-01-08

**Authors:** Jae-Ho Jeong, Kwangsoo Kim, Daejin Lim, Kwangjoon Jeong, Yeongjin Hong, Vu H. Nguyen, Tae-Hyoung Kim, Sangryeol Ryu, Jeong-A Lim, Jae Il Kim, Geun-Joong Kim, Sun Chang Kim, Jung-Joon Min, Hyon E. Choy

**Affiliations:** 1 Department of Microbiology, Chonnam National University Medical School, Gwangju, Republic of Korea; 2 Department of Nuclear Medicine, Chonnam National University Medical School, Gwangju, Republic of Korea; 3 Department of Biochemistry, Chosun University Medical School, Gwangju, Republic of Korea; 4 Department of Food and Animal Biotechnology, Seoul National University, Seoul, Korea; 5 School of Life Science, Gwangju Institute of Science and Technology (GIST), Gwangju, Republic of Korea; 6 Department of Biological Sciences, College of Natural Sciences, Chonnam National University, Yongbong-Dong, Buk-Gu, Gwangju, Korea; 7 Department of Biological Sciences, Korea Advanced Institute of Science and Technology, Daejeon, Korea; Charité-University Medicine Berlin, Germany

## Abstract

Bacterial cancer therapy relies on the fact that several bacterial species are capable of targeting tumor tissue and that bacteria can be genetically engineered to selectively deliver therapeutic proteins of interest to the targeted tumors. However, the challenge of bacterial cancer therapy is the release of the therapeutic proteins from the bacteria and entry of the proteins into tumor cells. This study employed an attenuated *Salmonella typhimurium* to selectively deliver the mitochondrial targeting domain of Noxa (MTD) as a potential therapeutic cargo protein, and examined its anti-cancer effect. To release MTD from the bacteria, a novel bacterial lysis system of phage origin was deployed. To facilitate the entry of MTD into the tumor cells, the MTD was fused to DS4.3, a novel cell-penetrating peptide (CPP) derived from a voltage-gated potassium channel (K_v_2.1). The gene encoding *DS4.3-MTD* and the phage lysis genes were placed under the control of *P_BAD_*, a promoter activated by L-arabinose. We demonstrated that DS4.3-MTD chimeric molecules expressed by the *Salmonellae* were anti-tumoral in cultured tumor cells and in mice with CT26 colon carcinoma.

## Introduction

In cancer therapy, the p53-induced apoptosis pathway, a major mechanism in tumor suppression, has been extensively exploited because of its role in the induction of apoptosis in cancerous cells [1], [2]. The deregulation of apoptosis leads to cancer development, proliferation, and treatment resistance [3], [4]. The mitochondria-mediated apoptotic pathway is largely regulated by the Bcl-2 family of proteins [5], which possess at least one of four conserved motifs known as the Bcl-2 homology domains (BH1 to 4). These domains have been divided into three subfamilies. One of these, the BH3-only proteins, including Bik, Bid, Bim, Bmf, Hrk, Bad, Puma, and Noxa, exhibit sequence homology only in the BH3 domain [5], [6]. Noxa is a transcriptional target of p53 that mediates induction of apoptosis via activation of mitochondrial damage and the intrinsic apoptosis signaling pathway [7], [8], [9], [10]. In recent studies, two functional domains in Noxa, the BH3 domain and the mitochondrial targeting domain (MTD), have been identified. Interestingly, the MTD was identified to be a prodeath domain, and shown to cause massive necrosis *in vitro* through cytosolic calcium increase; it is released from the mitochondria by opening the mitochondrial permeability transition pore [10], [11].

Bacterial cancer therapy relies on the fact that several bacterial species preferentially accumulate and replicate within tumors [12], [13], [14], [15]. In particular, several avirulent *Salmonella typhimurium* have been shown to be capable of reducing tumor mass [16], [17], [18], [19]. This trait of bacteria could be enhanced by genetic engineering aimed towards enabling these bacteria to express therapeutic molecules selectively in the targeted tumors. The therapeutic molecules, however, do not readily pass through bacterial membrane. Various methods, such as active transport of recombinant proteins by fusion with signal peptides, have been used to enable bacteria to secrete foreign proteins [20], [21]. One obvious limitation associated with this method is that secretion using signal peptides depends on the heteroprotein properties. To avoid these problems, a phage lysis system, most notably that of *E. coli* bacteriophage lambda, has been developed and applied to release heteromacromolecules of any kind [22], [23], [24]. Phage lambda encodes two proteins (S and R) which are sufficient to cause bacterial cell lysis [25], [26], [27]. Here, we employed lysis genes of a newly characterized *Salmonella* phage to release the heteromacromolecules from anti-tumoral *Salmonella typhimurium* [16], using MTD as an anti-tumoral cargo protein. This system was tested *in vitro* using cultured tumor cells and *in vivo* using tumor-bearing mice. Although the system was set up to express and release the therapeutic cargo out of the bacteria into the tumor tissue, the cargo also must subsequently be able to enter tumor cells. To overcome this problem, we constructed an MTD fused to a cell-penetrating peptide (CPP). CPPs are short, membrane-permeable, cationic peptides that are capable of both targeting intracellular proteins and carrying cargo proteins into tumor cells [28], [29], [30], [31]. The best studied CPPs include those derived from the trans-acting activator of transcription (TAT) protein from the human immunodeficiency virus 1 (HIV-1) [32] and those derived from penetratin, a Drosophila Antennapedia homeoprotein [33]. DS4.3 (RIMRILRILKLAR) is a newly identified CPP peptide derived from S5 subunit of a voltage-gated potassium channel (K_v_2.1) (see below, manuscript in preparation [34], [35]).In the present study, the DS4.3-MTD chimeric protein deployed by an attenuated *Salmonella typhimurium* was found to be effective both *in vitro* and *in vivo* against experimental tumor models.

## Materials and Methods

### Bacterial strains

The bacterial strains, all derived from wild-type *Salmonella typhimurium* 14028s, are listed in Table 1. ΔppGpp *S. typhimurium*, SHJ2037 (Δ*relA::km*, Δ*spoT::cm*), and SMR2130 (Δ*relA*, Δ*spoT*) have been previously described [36]. SKS001 (*Δglms::km*, [37]) and the other strains were constructed according to the method developed by Datsenko and Wanner [38]. For the imaging of bioluminescence, the whole luciferase operon of *Photorhabdus luminescens* from *S. typhimurium*-Xen26 (Caliper Life Sciences, USA) was transduced into strain SKS002 by P22HT int transduction [13], [39].

**Table 1:** Bacterial strains and plasmids used in this study.

Strains	Description	Reference
ATCC 14028s	Wild type *Salmonella typhimurium*	[59]
SHJ2037	ATCC 14028s, Δ*relA*::km, Δ*spoT::cm*	[36]
SMR2130	ATCC 14028s, Δ*relA*, Δ*spoT*	Laboratory stock
SHJ2168	ATCC 14028s, Δs*poT*, Δ*relA, lux::km*	[19]
SKS001	ATCC 14028s, Δ*glms::km*	[37]
SKS002	SKS001, Δ*relA*, Δ*spoT*, Δ*glmS::km*	[37]
SKS003	SKS002, *lux::km*	[37]

Plasmids	Description	Reference
pLYS	pBAD24 carrying phage lysislocus under *P_BAD_* and balanced lethal system with *glmS*	GenBank JF304023∼5
pBAD24*P_BAD_*::*lacZ*	*P_BAD_*::*lacZ* in pBAD24	This study
pLYS *P_BAD_*::*lacZ*	pLYS carrying *lacZ* under control of *P_BAD_*	This study
pLYS *P_BAD_*::*DS4.3-MTD*	pLYS carrying synthetic DNA for DS4.3 fused to human Noxa MTD under control of *P_BAD_*	This study

### Plasmids

The pLYS plasmid carried a 1.7 KB DNA fragment of the iEPS5 phage carrying lysis genes that had been cloned between the *NheI* and *EcoRI sites* in pBAD18 [40]. The pLYS plasmid and other plasmids used in this study carried the *glmS* gene from *Salmonella typhimurium* 14028s at the *ClaI* site in pBAD24. The *glmS* DNA was PCR-amplified using a pair of primers that contained the *ClaI* enzyme site and priming sequence with *glmS*: forward, ggatcgatatgtgtggaa ttgttggcgc, and reverse, ggatcgatttactctacggtaaccgatt (*ClaI* sites are underlined). pBAD24*P_BAD_*::*lacZ* was constructed by cloning the *lacZ* gene such that it was under the control of P_BAD_, as follows. The *lacZ* DNA was PCR amplified from pRS415 [41] using the following primers: forward, aagaattcgtcgttttacaacgtcgtga, and reverse, ttgtcgacttatttttgacaccagacca, carrying *EcoRI* and *SalI* sites (underlined), respectively, and cloned between the two restriction sites of pBAD24 [40]. The pLYS*P_BAD_*::*lacZ* plasmid was constructed by subcloning *lacZ* under the control of the P_BAD_ system into pLYS in the *PsiI* site using PCR amplification by primers carrying *SmaI* sequences and P_BAD_::*lacZ* priming sequences, with the pBAD24*P_BAD_*::*lacZ* as the template.

The DNA of human Noxa with a DS4.3 cell penetration peptide sequence (cgcattatgcgtattctgcgcattctgaaactggcgcgt) was synthesized and cloned under *P_BAD_* [40] between the *NcoI* and *PmeI* sites in pBAD24. The *DS4.3-MTD* with *P_BAD_* system was PCR-amplified using the following pair of primers, containing *SmaI* enzyme sites, with *P_BAD_::DS4.3-MTD* as a template: forward, aacccgggttatgacaacttgacggcta; reverse, aacccgggttatcaggttcctgagcaga (restriction sites are underlined). The amplified PCR product was digested with *SmaI* and ligated into the *PsiI* site of pLYS to generate pLYS*P_BAD_::DS4.3-MTD.*

### Growth conditions

Except when indicated otherwise, cultures were grown in LB medium (Difco Laboratories) containing 1% NaCl with vigorous aeration at 37°C. For solid support medium, 1.5% granulated agar (Difco Laboratories) was included. Antibiotics were from Sigma Chemical. When present, antibiotics were added at the following concentrations: ampicillin, 50 µg/ml; kanamycin, 50 µg/ml; chloramphenicol, 15 µg/ml.

### β-galactosidase assay

β-Galactosidase assays were performed essentially as described by Miller [42]. For determinations of β-galactosidase levels in bacteria under different conditions of induction and non-induction, overnight cultured bacteria were diluted 1∶50 in 50 ml LB media as described in the text. Cultures were further incubated at 37°C to log phase and induced with L-arabinose to a final concentration of 0.04%. The cultures were taken at the indicated times and were separated into supernatant and bacterial pellet fractions by centrifugation (3000× g, 10 min) and filtration (0.45 µm). Each sample was assayed in triplicate.

### Cell lines and animals

Five- to six-week-old male Balb/c mice were purchased from Samtako Korea. CT26 mouse colon cancer cells, HeLa cells, and Hep3B cells, from the Waterborne Virus Bank (Seoul, Korea), were grown as a monolayer in Dulbecco's modified Eagle's medium (DMEM) supplemented with 10% FBS and 1% antibiotic-antimycotic mixture (Gibco, Invitrogen). Tumor-bearing mice were generated by subcutaneous implantation of CT26 tumor cells (10^6^ cells suspended in 50 µl PBS) on the right thigh. After about 10–15 days, the tumor sizes reached approximately 100–150 mm^3^ and they were used for the experiments. The animal studies were carried out under standard animal welfare conditions and were approved by the Chonnam National University Animal Care and Use Committee (accession number: CNU IACUC H-2013-3).

### SDS-PAGE and Western blot analysis

For visualization of β-galactosidase and Noxa by Western, the cultures were taken at the indicated times and were separated into supernatant and bacterial pellet fractions by centrifugation and filtration. The samples were sonicated in the presence of SDS (0.1%) and protein concentrations were determined by Bradford method [43]. 50 µg of each sample was loaded on 8–15% SDS-PAGE gels and transferred to a nitrocellulose membrane (Bio-Rad, Hercules, CA, USA). After the transfer, the membrane was blocked with 5% skim milk and probed with 1∶10,000-diluted mouse anti-β-galactosidase antibody (Santa Cruz, sc-65670) or 1∶500-diluted rabbit anti-Noxa BH3 domain antibody (ABGENT, AP1316a) at 4°C overnight. Subsequently, the membrane was incubated with goat anti-mouse IgG-linked horseradish peroxidase (Santa Cruz, sc-2005) or goat anti-rabbit IgG-linked horseradish peroxidase (Santa Cruz, sc-2004) under the same conditions for 1 hr.

### Optical bioluminescence imaging

Bioluminescence imaging was performed as previously described using an IVIS 100 system (Caliper) [13].

### Cell death assay

The survival rate of the cancer cells was determined by 3-(3,4-dimethylthiazol-2-yl)-2,5-diphenyltetrazolium bromide (MTT) assay as described [44]. Each cell line was seeded into 96-well plates at 10,000 viable cells per well. Twenty-four hours later, the cells were washed with PBS and placed in serum-free medium containing varying concentrations of chimeric Noxa (1, 10, 20, and 50 µM) or concentrated supernatants from bacterial lysates (the protein concentrations were 2, 20, and 200 µg). The filtrated bacterial supernatants was concentrated with a centrifugal filter (Amicon® Ultra-15, 3K), and the protein concentrations were determined by Bradford method [43]. Twenty-four hours later, the surviving cells were stained with MTT and quantified by absorbance at 540 nm. The MTT assay results were plotted with mean ± S.D. of three experiments.

### Histochemical and immuno-fluorescence Stain

Tumor tissue was collected from experimental mice that had undergone bacterial therapy and was embedded into an O.C.T. compound (Sakura, USA). Cryosections of 7 µm were obtained and mounted on ProbeOnTM Plus microscope slides (Fisher, USA). The β-Galactosidase Stain was performed with a β-Galactosidase Staining Kit (Mirus Bio LLC, USA). Briefly, the slides were washed with PBS and incubated in X-gal (5-bromo-4-chloro-indolyl-β-D-galactopyranoside) staining solution at 37°C for 4 hrs in a humidified chamber, counter-stained with hematoxylin, and mounted with Faramount (Dako, USA). For immuno-fluorescence Stain, frozen section was fixed with cold acetone and Noxa was detected by rabbit anti-Noxa IgG (SantaCruz, SC-30209) and goat anti-rabbit IgG-FITC (SantaCruz, SC-2012) and nuclei was stained with 4,6-diamidino-2-phenylindole (DAPI). Stained samples were mounted using the SlowFade/Antifade Kit (Invitrogen, Carlsbad, CA, USA). The stained slides were examined using an Olympus BX51 microscope and an imaging program (analySIS LS starter).

### Statistical analysis

Statistical analysis was performed using the SPSS 18.0 statistical package (SPSS Inc., Chicago, IL, USA). Statistical analysis was performed using the two-tailed Student's *t* test or two-way ANOVA. A *P* value of <0.05 was considered statistically significant (* P<0.05, *** P<0.0001).

## Results

### Characterization of bacterial lysis induced by a lysis system of novel phage origin

**Figure 1:**
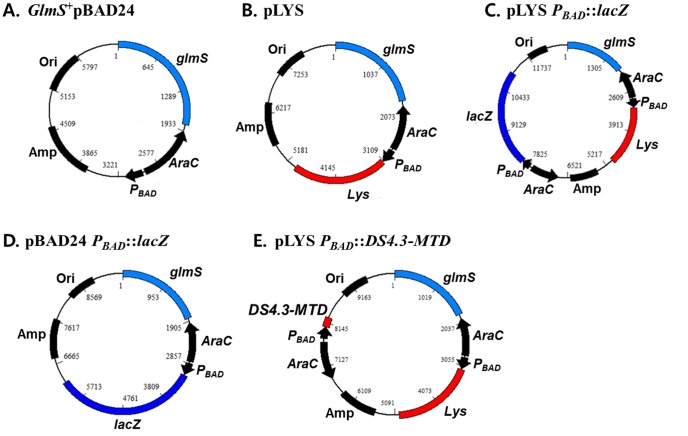
Schematic diagram of the plasmids used in this study. ‘Lys’ carried the locus with *Salmoella* lysis function of the phage iEPS5 phage.

In an attempt to develop a system of delivery of anti-tumoral MTD into tumor tissue through *Salmonellae*, a bacterial lysis system of *Salmonella* phage origin was examined. The DNA of the iEPS5 phage, isolated from a field sample, was partially digested with the restriction enzymes *NheI* and *EcoRI*. Each of the fragments was then placed under the control of the *P_BAD_* promoter of the *E. coli* arabinose operon, which is induced by L-arabinose, and cloned into pBAD24 [40]. *S. typhimurium* 14028s was transformed with the recombinant plasmids and the library was examined for the lysis phenotype. The clones that showed the lysis phenotype upon addition of L-arabinose were selected. By sequencing the inserted fragments, a plasmid carrying a 1,856 bp DNA fragment (GenBank JF304023∼5) with the lysis phenotype was selected and named ‘*Lys*’ [45] (Fig. 1). For subsequent studies, we used the highly attenuated ΔppGpp *S. typhimurium* [36], which also carries a mutation in the *glmS* gene, as the host. Thus, all the plasmids used in this study carried *glmS* (Fig. 1A, *GlmS^+^p*) so that the plasmids were maintained in the absence of selection pressure [37]. This balanced lethal host vector system using *GlmS^+^p* relies on the phenotype of the GlmS^−^ mutant, which undergoes lysis when grown in the absence of N-acetyl-D-glucosamine (GlcNac) [37], [46], [47], [48]. The ‘*Lys*’ fragment was subcloned into pBAD24 carrying *glmS* gene to generate pLYS (Fig. 1B).

**Figure 2:**
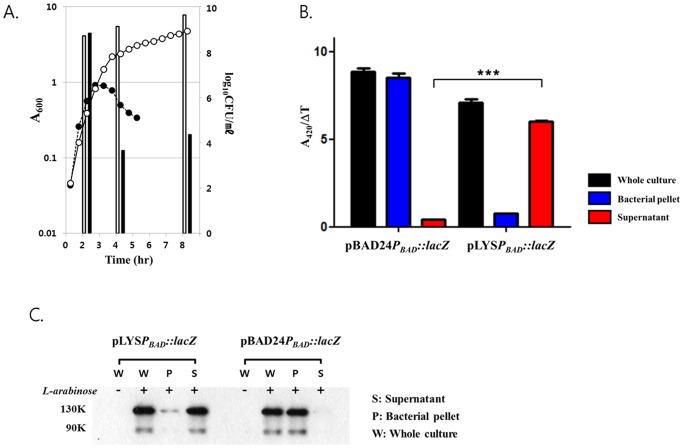
Bacterial lysis phenotype of pLYS. (A) *S. typhimurium* carrying pLYS were grown in LB supplemented with L-arabinose. When the optical density (A_600_) of the culture reached ∼1.0, at T = 2 hrs, the LB was supplemented with L-arabinose in half of the cultures. The A_600_ of the culture was measured at regular time intervals (circles) and viable bacteria were determined by CFU counting (bars). Open circles and bars represent bacteria samples without L-arabinose addition, and filled circles and bars represent bacterial samples with L-arabinose addition. (B) *S. typhimurium* carrying either pLYS*P_BAD_::lacZ* or pBAD24*P_BAD_::lacZ* grown in LB was supplemented with L-arabinose when the A_600_ of the culture reached ∼1.0. The bacterial culture was taken 2 hrs after the addition of L-arabinose, separated into bacterial pellet and supernatant by centrifugation and filtration, and assayed for total β-galactosidase activity. Data represent mean ± S.D., and asterisks (*) indicate a significant difference compared between supernatant of pLYS*P_BAD_::lacZ* and pBAD24*P_BAD_::lacZ* (***, P<0.0001). (C) The same samples were analyzed for β-galactosidase expression by Western blot analysis. 50 µg of sonicated samples were analyzed. Bacterial whole culture not induced for bacterial lysis was included.

First, the phenotype of controlled expression of the putative lysis function of pLYS was examined under *in vitro* growth conditions (Fig. 2A). *S. typhimurium* defective in ppGpp synthesis carrying pLYS was grown in LB. When the A_600_ of the culture reached ∼1.0, at T = 2 hrs, L-arabinose was added to a final concentration of 0.04% (w/v), and the optical density of the culture (A_600_) and total colony-forming units (CFU) were determined at the indicated times. The culture continued to proliferate in the absence of L-arabinose addition, up to more than 5×10^9^ CFU/ml and A_600_ ∼5.0 at T = 4 hrs and thereafter. However, upon addition of L-arabinose to the culture, CFU reduced about 10^5^-fold, to about 1×10^4^ CFU/ml and an A_600_<0.5 at T = 4 hrs. The optical density of the culture (A_600_) changed accordingly: decreased upon addition of L-arabinose while increased in its absence. This result demonstrated the lysis function of the ‘*Lys*’ fragment cloned in pLYS, and also the control of its expression by L-arabinose.

Subsequently, the nature of the bacterial cell death following expression of the lysis genes was determined *in vitro* to verify that the lysis had resulted in the release of cellular contents into the media. SKS002 (ΔppGpp, Δ*glmS::km*) was transformed with pLYS*P_BAD_*::*lacZ* (Fig. 1C) or pBAD24*P_BAD_*::*lacZ* carrying *lacZ* (Fig. 1D) encoding β-galactosidase under the control of the *P_BAD_* promoter. The *Salmonellae* transformed with the pLYS*P_BAD_*::*lacZ* expressed both lysis protein and β-galactosidase, and those transformed with the pBAD24*P_BAD_*::*lacZ* only expressed β-galactosidase, upon addition of L-arabinose. When the A_600_ of the culture reached ∼1.0, L-arabinose was added and samples were taken 2 hrs after the induction. Supernatant was separated from cultured bacteria by centrifugation and filtration. The filtered culture media, bacterial pellet, and whole culture were examined for β-galactosidase activity [42]. As expected, in the case of *Salmonellae* carrying the pLYS*P_BAD_*::*lacZ* strain, over 90% of the β-galactosidase activity was detected in the filtered culture media. By contrast, β-galactosidase activity in the *Salmonellae* carrying *P_BAD_*::*lacZ* was detected mainly in the bacterial pellet (Fig. 2B). This was further verified by Western blot analysis using a β-galactosidase-specific antibody with the same samples (Fig. 2C). β-galactosidase-specific bands (∼130 kDa) were detected in the whole-culture media of *Salmonellae* carrying either plasmid only upon L-arabinose addition. Most importantly, for the *Salmonellae* carrying pLYS*P_BAD_*::*lacZ*, almost the same amount of β-galactosidase band was detected in the filtered media as in the whole culture, and little was detected in the bacterial pellet. Conversely, for the *Salmonellae* carrying pBAD24*P_BAD_*::*lacZ*, the majority of the β-galactosidase was found in the bacterial pellet and little was detected in the filtered media. Taken together, this suggested that the majority of the proteins encoded in pLYS*P_BAD_*::*lacZ* were released into the medium upon induction of the lysis function.

### Control of lysis system in vivo

**Figure 3:**
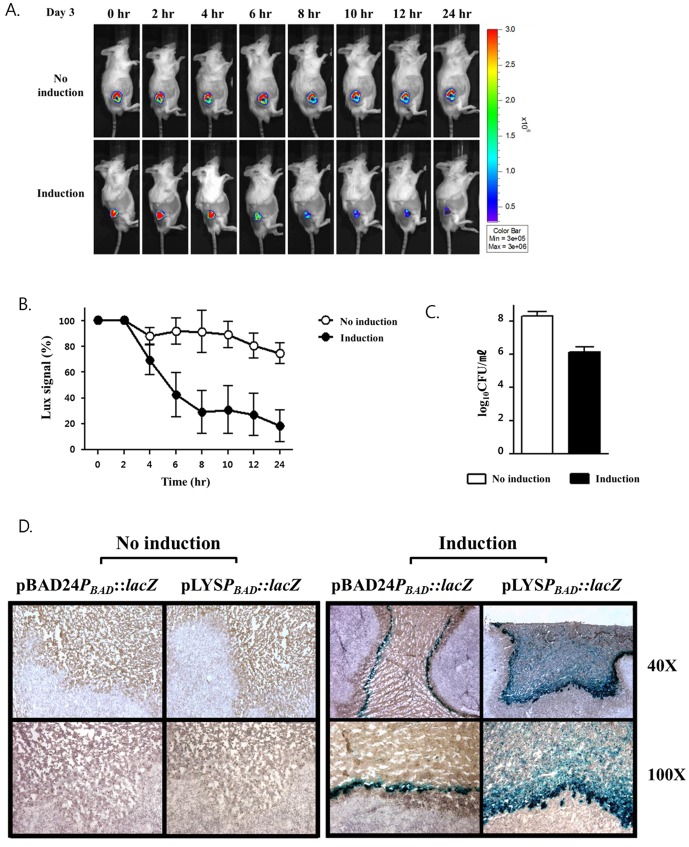
Monitoring of L-arabinose-induced bacterial lysis by a non-invasive *in vivo* imaging system (IVIS) and release of β-galactosidase from *Salmonellae* carrying pLYS*P_BAD_*::*lacZ* or pBAD24*P_BAD_::lacZ* targeted to implanted tumor tissue (CT26) in BalB/c mice. Each time point represents the mean for n = 3 animals. (A) Bioluminescent bacteria (SKS003) carrying pLYS were injected intravenously into CT26 tumor-bearing mice. After three days, the bioluminescence signal from the *Salmonellae* was monitored by a non-invasive *in vivo* imaging system (IVIS) at the indicated times after intraperitoneal injection of L-arabinose (60 mg) in the induced group. The Y-axis indicates photons ×10^5·^s^−1·^cm^−2·^sr^−1^. The ROI was selected manually for quantification of photon flux. The ROI area was kept constant, and the intensity was recorded as a maximum (photons ×10^5·^s^−1·^cm^−2·^sr^−1^) within the ROI. (B) Photons from each mouse at 0 time was calculated 100% and compared. Data represent mean ± S.D., and asterisks (*) indicate a significant difference compared between uninduced and induced group (***, P<0.0001). (C) The number of live bacteria in the tumor tissue 8 hrs after the addition of L-arabinose was determined by CFU counting. (D) The tumor tissues were analyzed for the expression and distribution of β-galactosidase by histochemical staining with X-gal.

Next, we examined the controlled expression of the lysis system carried by *S. typhimurium* targeted to a tumor mass grafted in mouse model. This was because MTD is a highly cytotoxic protein that cannot be constitutively released without significant damage to nontumor tissue [10]. Thus, we determined biodistribution of the ΔppGpp *Salmonella* 3 days post infection (dpi) of BALB/c mice ectopically implanted with mouse CT-26 colon carcinoma cells, via intravenous route. Bacteria were counted in two major reticuloendothelial organs, liver and spleen, in addition to tumor tissue by CFU determination (Table 2). The results demonstrate that ΔppGpp *Salmonella* preferentially accumulated in tumors over livers at a ratio of >48,000 : 1, and over spleen at a ratio of >65,000 : 1. All tumor-to-liver ratios were statistically significant (*P*<0.05). Previously, we have analyzed clinical parameters of CT26-bearing mice treated with the ΔppGpp *Salmonella* carrying cytolysin A (Cly A) under the control of inducible promoter, *TetA* promoter [49]. The analysis revealed a significant elevation of the markers of inflammation and hepatic function in the animals that were induced at 0 dpi but not in those induced at 3 dpi. Taken together, we concluded that it would be safe to induce lysis of intratumoral *Salmonellae* at 3 dpi. To monitor intratumoral bacterial lysis, we used *Salmonellae* carrying a bacterial luciferase (lux) operon in their chromosome [19], that enables non-invasive imaging of live bacteria using *in vivo* optical imaging system. These bioluminescent bacteria (SKS003), carrying pLYS, were injected intravenously into CT26-bearing (Fig. 3A). L-arabinose (60 mg) was administrated to animals by intraperitoneal injection at 3 dpi. As shown in Figure 3A, the bioluminescent signal decreased considerably compared with uninduced controls approximately 8 hrs after the administration of L-arabinose (Fig. 3A). Figure 3B shows the quantification of this result. At last, the number of live bacteria in the tumor tissue 8 hrs after the induction of the lysis system was determined by counting the CFUs from homogenized tumor tissues (Fig. 3C). Bacterial CFUs had decreased by over 99% at 8 hrs after L-arabinose administration, compared with the uninduced control group. This result coincided with the decreased bioluminescence signals seen after administration of L-arabinose in the tumor site (Fig. 3A and B).

**Table 2:** Biodistribution of ΔppGpp *Salmonella typhimurium.*

cfu/g Tumor (range)	cfu/g Liver (range)	cfu/g Spleen (range)	Ratio (Tumor : organ)
1,500,542,100 (0.2∼3.7×10^9^)	30,722 (1.3∼6.3×10^4^)	22,765 (0.8∼6.6×10^4^)	48,842 : 1 (liver) 65,941 : 1 (spleen)

Mice (n = 5) were implanted subcutaneously with CT26 cells. 1×10^7^ ΔppGpp *Salmonellae* were injected intravenously when the tumors reached 100∼150 mm3. After 3 days, liver, spleen, and tumor were collected and analyzed for CFU determination.

The release of the cytosolic contents of bacteria following induction of the lysis system was further verified in the mouse model using *Salmonellae* expressing β-galactosidase. The mice bearing CT26 were intravenously injected with *Salmonellae* (SKS002) carrying either pLYS*P_BAD_*::*lacZ* or pBAD24*P_BAD_*::*lacZ*. Three days after the bacterial injection, L-arabinose was administrated by intraperitoneal injection. Eight hours later, tumor samples were taken from the mice and the distribution of β-galactosidase following induction of bacterial lysis was analyzed by histochemical staining of the tumor tissue samples with X-gal (Fig. 3D). Blue pigmentation from β-galactosidase was detected in mice treated with *Salmonellae* carrying either plasmid, but only after L-arabinose administration. It should be noted that the scattering of blue pigmentation widely out of the area targeted by the *Salmonellae* carrying pLYS*P_BAD_*::*lacZ*, but not in the tumor tissue targeted by the *Salmonellae* carrying pBAD24*P_BAD_*::*lacZ.* In the latter case, the blue pigmentation was confined to the narrow area between necrotic and proliferative regions where *Salmonellae* were known to be localized [50], [51]. Taken together, this was interpreted to mean that β-galactosidase induced in *Salmonellae* targeted to the tumor by L-arabinose, was released from the *Salmonellae* upon induction of the lysis system, and spread into neighboring areas, necrotic and proliferative tumor regions. Thus, the phage lysis system would be applicable to deliver proteinous antitumor cargo into tumor tissue.

### Antitumor effect of the MTD

**Figure 4:**
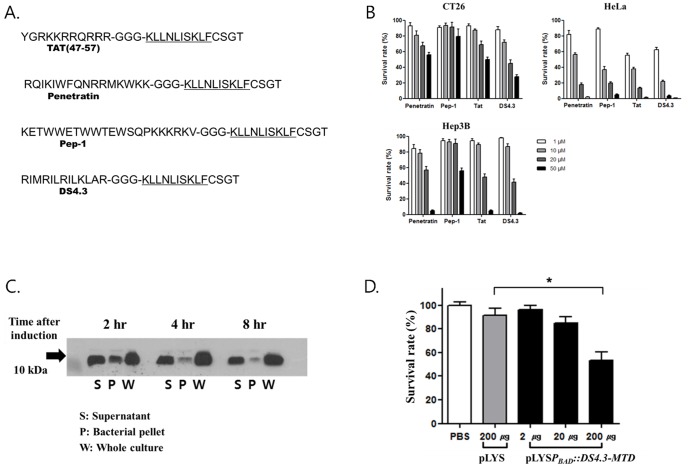
Induction of cell death by MTD fused to CPPs. (A) Amino acid sequences of chimeric CPP-MTDs used in this study. The CPP-MTDs were arranged as follows: N′ CPP followed by a GGG linker, KLLNLISKLF (the MTD sequence), and finally the CSGT of the C-terminal end of Noxa [10]. (B) Induction of cell death by CPP-MTD peptides as assessed by MTT assay. *In vitro* cultured HeLa, Hep3B, and CT26 cells were tested. (C) The expression and release of DS4.3-MTD from the *Salmonellae* carrying pLYS*P_BAD_*::*DS4.3*-*MTD* at indicated time after L-arabinose addition, as analyzed by Western blot using anti-Noxa antibody. The samples were prepared as described in the legend of Figure 2. (D) Induction of cell death by the bacterial supernatant of *S. typhimurium* carrying pLYS*P_BAD_*::*DS4.3*-*MTD* after L-arabinose addition. Bacterial supernatant of *S. typhimurium* carrying pLYS after L-arabinose addition was included. Data represent mean ± S.D., and asterisks (*) indicate a significant difference compared between pLYS and pLYS*P_BAD_*::*DS4.3*-*MTD* (*, P<0.05).

Although the system was set up to express and release the MTD from the *Salmonellae* into the tumor tissue, the peptide needs to get into the tumor cells to be effective. The MTD by itself cannot penetrate tumor cells; however, the MTD fused to eight arginine residues (R8-MTD) has been shown to be able to penetrate into tumor cells and induce necrotic cell death [10]. In this study, therefore, we fused the MTD to different CPPs to improve the delivery into tumor cells further [29], [30]. We synthesized peptides consisting of the eleven amino acids of the MTD and four C′ terminal amino acids fused to the C′ terminus of four kinds of CPPs with a trimeric glycine linker: TAT of HIV, penetratin of Drosophila, and, DS4.3 of K_V_2.1.Pep-1 is an artificial CPP [52] (Fig. 4A). The CPP-MTD peptides were synthesized and tested for their abilities to induce cell death *in vitro* using various cell lines. We added 0–50 µM chimeric CPP-MTD peptides into Hep3B, HeLa, and CT26 cells (Fig. 4A and B). After 24 hrs incubation, cell survival was determined by MTT assay. In all cases, the treatment with chimeric CPP-MTD caused cell death in a dose-dependent manner. However, DS4.3-conjugated MTD induced cell death at the lowest concentration among all of the chimeric CPP-MTD peptides tested (Fig. 4B). Thus, the 30-amino acid DS4.3-MTD chimeric peptide was chosen as the cargo to be delivered by the *Salmonellae* using the phage lysis system. The DNA encoding the nucleotide sequence for *DS4.3-MTD* was synthesized and cloned under the L-arabinose-inducible *P_BAD_* in the pLYS plasmid background, generating pLYS*P_BAD_*::*DS4.3-MTD* (Fig. 1E). *Salmonellae* (SK2002) transformed with this plasmid were cultured in LB to an A_600_ of about 1.0 and induced for the expression of DS4.3-MTD as well as the phage lysis system by the addition of L-arabinose. Samples of the bacterial culture were taken at the indicated times after the induction, and the media and bacterial pellets were divided, and analyzed for DS4.3-MTD expression by Western blot using an MTD-specific antibody (Fig. 4C). Two hours after induction with L-arabinose, DS4.3-MTD was detected in the supernatant media as well as in the bacterial pellet. Four and 8 hrs after the induction, DS4.3-MTD was detected mainly in the supernatant, suggesting that the lysis system functioned properly in this setting.

Subsequently, the DS4.3-MTD expressed in the *Salmonellae* and released into the media was tested for its cytotoxic effect on tumor cells (Fig. 4D). The supernatant of the culture taken after 4 hrs of induction was concentrated, added to cultured CT26 cells, and incubated overnight. Cell death was determined by MTT assay. The concentrated supernatant media of the *Salmonellae* carrying pLYS*P_BAD_*::*DS4.3*-*MTD* induced with L-arabinose showed the induction of cell death in a dose-dependent manner. By contrast, the concentrated supernatant of the *Salmonellae* carrying pLYS showed no cell death effect. Thus, DS4.3-MTD expressed and released by *Salmonellae* was demonstrated to be effective *in vitro.*

**Figure 5:**
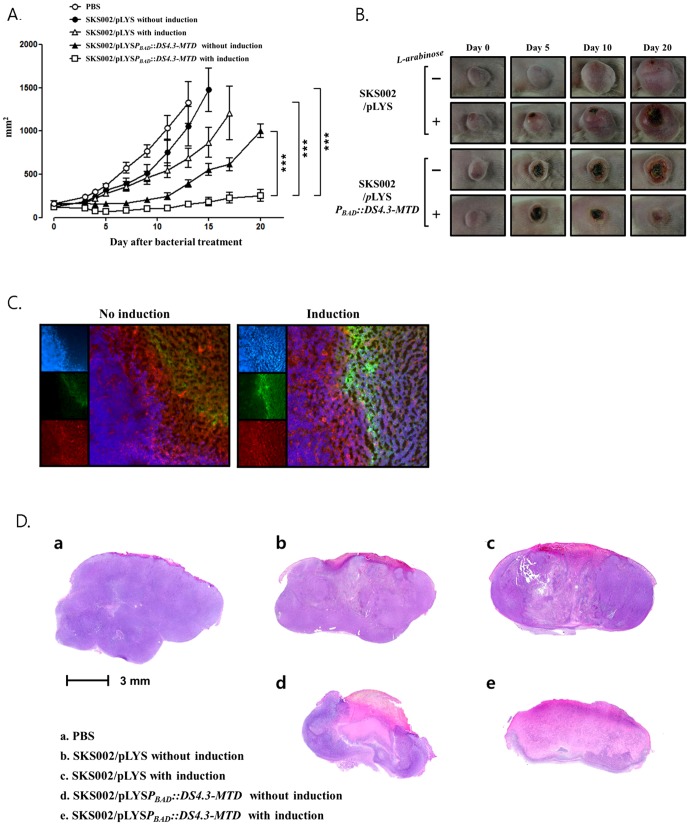
Anti-tumoral effects of Salmonellae carrying either pLYSP_BAD_::DS4.3-MTD or pLYS, Three days after the bacterial treatment, L-arabinose (60 mg) was intraperitoneally injected daily (n = 3 per group). (A) Changes in tumor size after the bacterial treatment. Data represent mean ± S.D., and asterisks (*) indicate a significant difference compared between each groups with two-way ANOVA (***, P<0.0001) (B) A representative morphological change of the implanted CT26 tumor after the bacterial treatment. (C) Immuno-fluorescence examination of CT26 tumor tissue after bacterial treatment. Frozen section was obtained from the tumor tissue and stained with Noxa specific antibody (SantaCruz, sc-30209). The boxes on left show nucleus with DAPI in blue, Noxa MTD with specific antibody in green, and cytosol with TexasRed Phalloidin in red, in order from the top. The boxes on the right show merged images. (D) Histopathological examination of CT26 tumor tissue after the bacterial treatment. Tumor tissues were sliced and stained with hematoxylin-eosin (HE). Purple area represents the region with healthy cells and whitish pink the region with anucleus dead cells, the necrotic region.

Lastly, the antitumor effect of the *Salmonellae* expressing DS4.3-MTD was tested using BALB/c mice implanted with CT26 on the right thigh. A total of 1×10^7^ *Salmonellae* (SK002) carrying pLYS or pLYS*P_BAD_*::*DS4.3-MTD* were injected into the tumor-bearing mice through the tail vein when the tumor size reached about 100–150 mm^3^. Three days after the injection, L-arabinose was intraperitoneally injected and changes in the size of the tumor were determined (Fig. 5A and B). The results showed a maximum anti-tumoral effect with *Salmonellae* carrying pLYS*P_BAD_*::*DS4.3-MTD* induced by L-arabinose injection, followed by the same treatment without L-arabinose injection. We also observed an antitumor effect from the *Salmonellae* carrying pLYS induced for bacterial lysis, and this effect was considerably greater than that without bacterial lysis, which was about the same as that in untreated controls (the PBS-treated group).

Subsequently, we verified the expression and release of DS4.3-MTD in the above tumor tissue upon administration of L-arabinose (Fig. 5C). Immuno-fluorescence staining revealed expression of DS4.3-MTD in the induced samples at the area between necrotic and proliferative regions where *Salmonellae* were known to be localized [50], [51]. Evidently, DS4,3-MTD under the control of *P_BAD_* was induced as well as β-galactosidase (Fig. 3D) in the mouse model upon administration of L-arabinose. However, we observed the presence of MTD in the uninduced samples, although marginal, suggesting that DS4.3-MTD expressed at the basal level would be responsible for the anti-tumoral effect with *Salmonellae* carrying pLYS*P_BAD_*::*DS4.3-MTD* without L-arabinose injection (Fig. 5A). The tumors from the mice treated above were extracted 5 days after the L-arabinose administration and analyzed by H&E staining (Fig. 5D). This histopathological analysis revealed that the cells died by necrosis. The extent of the necrosis was most serious in the tumor samples taken from mice treated with bacteria carrying pLYS*P_BAD_*::*DS4.3-MTD* induced by L-arabinose administration (over 70%), and less so in those from uninduced mice and those from mice treated with *Salmonellae* carrying pLYS, following the order of antitumor effects of the treatments. Taken together, using a mouse model, the DS4.3-MTD expressed and released from *Salmonellae* by the phage lysis system was demonstrated to be effective in inducing necrosis of tumor cells, resulting in tumor suppression.

## Discussion

The MTD has been shown to induce necrotic cell death indiscriminately both in tumor cells and normal cells [11]. Thus, unless a specific tumor-targeting signal is employed [10], MTD cannot be developed as an antitumor drug. Our bacterial delivery system provides an alternative means to deliver the MTD of Noxa selectively to the tumor site, by taking advantage of tumor-targeting characteristics of the bacteria. However, the delivery of therapeutic materials requires release of the macromolecules from the bacteria after colonization of tumor tissue. Various bacteria delivery systems have been developed for this purpose. Although selective secretion using a signal peptide would be one way to overcome the barrier, finding a suitable signal peptide for a specific macromolecule is a cumbersome process. Alternatively, macromolecules can be released from bacteria by the lysis of the bacterial membrane with a phage lysis system as demonstrated in this study. The advantage of using this type of lysis system is that macromolecules of any kind would be delivered in this way into the tumor site; however, this requires controlled expression of the lysis gene so that the system will be turned on only when bacteria have predominantly accumulated in the tumor. An obvious disadvantage, however, using one inducible promoter, *P_BAD_* in this case, for induction of both tumorlytic gene and the lysis gene, would be sub-maximal expression of the tumorlytic gene, which might consequently diminish the antitumoral therapeutic effect. Thus, it would be desirable to use different inducible promoter systems, *i.e.* a combination of *tet* promoters [49] and *P_BAD_*, for induction of tumorlytic gene and the lysis gene.

Usually, 3–4 days after the injection of bacteria, we observed the greatest preferential accumulation of bacteria in tumors over endothelial organs. Interestingly, this ratio varied depending on the bacterial strains and model animals. We counted 0 CFU in the reticuloendothelial organs with *E. coli* (MG1655) in tumor-bearing mice [13]. With ΔppGpp *Salmonella*, we counted ∼10^4^ CFU, thus ratio of tumor-to-reticuloendothelial organs ranged in ∼10^5^ (Table 2), which was far better than the *Salmonella* VN20009, a widely used bacterial therapy strain, of which ratio ranged in ∼10^3^ [53]. Our recent publication reported biodistribution of ΔppGpp *S. typhimurium* in myocardial infarction (MI) rat models, in which ΔppGpp *Salmonella* selectively accumulated and proliferated in MI tissue [54]. Early after injection (12 hours), bacterial load was found primarily in the spleen and liver, most likely captured by phagocytic macrophages [55], with a relatively small bacterial burden in the heart. After 24 hours, however, the number of CFUs in myocardial tissue increased dramatically, reaching a maximum on 3 and 5 dpi of ∼10^6^ CFU/g, whereas the bacterial burden in the liver and spleen declined over the same period of time to undetectable levels. No bacterium was detected in the liver and spleen on 5 dpi. No sign of serious local or systemic inflammatory reactions was noted following i.v. administration of ΔppGpp *S. typhimurium*: the levels of c-reactive protein (CRP) and procalcitonin (PCT) were not significantly changed. A similar result was obtained with ΔppGpp *Salmonellae* carrying ClyA [49]. Thus, 3 dpi would be the time point to express and release anti-tumoral macromolecules from the bacterial strain used in this study (Fig. 3). The *P_BAD_* system has provided an efficient means of controlling gene expression of bacteria in tumors remotely by intraperitoneal administration of its inducer, L-arabinose [19], [56]. However, the evident basal level expression of the cargo proteins in the absence of inducer (Fig. 2C and 5C) demands further improvement of the regulatory circuit.

In most cancers, blood vessel growth does not keep pace with tumor cell growth that leads to pronounced hypoxia often accompanied by necrosis [57]. Facultative anaerobes such as *Escherichia coli* and *Salmonella spp.* should be able to grow in viable as well as necrotic areas of the tumor. Previously, we have traced the *E. coli* that was i.v. injected in model mice bearing CT26, and reported that bacteria were detected initially towards the center of the tumor. As time passed, they appeared to spread to the border between the necrotic and viable areas of the tumor. Concomitantly, β-galactosidase and MTD expressed from intratumoral *Salmonella* were found at the area between necrotic and proliferative regions where *Salmonellae* were known to be localized (Fig. 3D and Fig. 5C). The confinement of bacteria has been suggested to be due to host immune system that prevents bacterial dissemination throughout tumors [51]. In particular, neutrophils were shown to prevent bacteria from spreading from necrotic to viable tumor tissue. In fact, however, bacterial-induced destruction of viable cells would position bacteria at the interface with the necrotic region. At last, as tumor regresses, bacteria would be confined to the outermost part where scarcely any viable region remains. Consistently, a remarkable correlation between antitumor effect of bacterial treatment and the extent of necrosis was observed (Fig. 5D).

One of the main concerns raised regarding bacterial therapy is the possibility of infection caused by the bacteria used for the antitumor therapy. This, however, would not be a problem in this system, since the therapeutic bacteria are easily cleared using antibiotics, as demonstrated previously [54]. In this study, we showed that the *Salmonellae* equipped with the phage lysis system do undergo lysis upon its induction: over 99.9% of bacteria were lysed upon induction with L-arabinose *in vivo* and *in vitro* (Figs. 2A and 3C). In fact, we observed that at the end of the bacterial treatment with the phage lysis system (∼two to three weeks after the injection), the number of bacteria counted at the tumor site was less than 100 (manuscript in preparation). This is probably because bacteria are no longer protected in immunologically privileged environment [58], as tumor regresses, and thereby cleared by host immune system. Thus, this feature could provide another level of safety by reducing the number of live bacteria. Taken together, the phage lysis system provides not only a means to release antitumoral macromolecules from the bacteria, but also an additional level of safety for bacteria-mediated cancer therapy.
